# PBPK Modeling of Lamotrigine and Efavirenz during Pregnancy: Implications for Personalized Dosing and Drug-Drug Interaction Management

**DOI:** 10.3390/pharmaceutics16091163

**Published:** 2024-09-03

**Authors:** Bárbara Costa, Maria João Gouveia, Nuno Vale

**Affiliations:** 1 PerMed Research Group, Center for Health Technology and Services Research (CINTESIS), Rua Doutor Plácido da Costa, 4200-450 Porto, Portugal; b.c.211297@gmail.com; 2 CINTESIS@RISE, Faculty of Medicine, University of Porto, Alameda Professor Hernâni Monteiro, 4200-319 Porto, Portugal; 3 Department of Community Medicine, Health Information and Decision (MEDCIDS), Faculty of Medicine, University of Porto, Alameda Professor Hernâni Monteiro, 4200-319 Porto, Portugal; 4 Centre for Parasite Biology and Immunology, Department of Infectious Diseases, National Health Institute Dr. Ricardo Jorge, Rua Alexandre Herculano 321, 4000-055 Porto, Portugal; m.joao.gouveia@insa.min-saude.pt; 5 Center for Study in Animal Science of University of Porto (CECA-ICETA UP), Praça Coronel Pacheco 15, 4050-453 Porto, Portugal

**Keywords:** pharmacokinetics, pregnancy, PBPK models, lamotrigine (LTG), efavirenz (EFV), drug-drug interactions (DDI), UGT enzyme, therapeutic management

## Abstract

This study aimed to model the pharmacokinetics of lamotrigine (LTG) and efavirenz (EFV) in pregnant women using physiologically based pharmacokinetic (PBPK) and pregnancy-specific PBPK (p-PBPK) models. For lamotrigine, the adult PBPK model demonstrated accurate predictions for pharmacokinetic parameters. Predictions for the area under the curve (AUC) and peak plasma concentration (Cmax) generally agreed well with observed values. During pregnancy, the PBPK model accurately predicted AUC and Cmax with a prediction error (%PE) of less than 25%. The evaluation of the EFV PBPK model revealed mixed results. While the model accurately predicted certain parameters for non-pregnant adults, significant discrepancies were observed in predictions for higher doses (600 vs. 400 mg) and pregnant individuals. The model’s performance during pregnancy was poor, indicating the need for further refinement to account for genetic polymorphism. Gender differences also influenced EFV pharmacokinetics, with lower exposure levels in females compared to males. These findings highlight the complexity of modeling EFV, in general, but specifically in pregnant populations, and the importance of validating such models for accurate clinical application. The study highlights the importance of tailoring dosing regimens for pregnant individuals to ensure both safety and efficacy, particularly when using combination therapies with UGT substrate drugs. Although drug-drug interactions between LTG and EFV appear minimal, further research is needed to improve predictive models and enhance their accuracy.

## 1. Introduction

Pregnant women affected by epilepsy and HIV often need to take medication. The pharmacokinetics of antiretroviral and anticonvulsant drugs can significantly impact physiological changes during pregnancy and drug-drug interactions (DDIs) [1,2]. Adequate exposure to antiretroviral drugs (ARVs) is crucial for achieving and maintaining virologic suppression in patients living with HIV [3,4]. However, pregnancy introduces complex changes in drug metabolism, absorption, and elimination, necessitating careful consideration when selecting ARV regimens [5,6]. ARVs such as protease inhibitors, non-nucleoside reverse transcriptase inhibitors, and the integrase strand inhibitor elvitegravir are extensively metabolized by the cytochrome P450 (CYP450) enzyme system, leading to clinically relevant DDIs [7,8]. For instance, CYP induction by rifampicin decreases ARV exposure [9], while CYP3A4 inhibition by ritonavir or cobicistat increases it [10,11,12]. These interactions extend beyond CYP metabolism to involve non-CYP pathways, drug transporters, and pharmacokinetic processes.

Additionally, pregnancy alters drug metabolism through changes in gastric pH, intestinal motility, plasma protein concentrations, hepatic blood flow, metabolic enzyme activity, glomerular filtration rate, and renal blood flow [7,13]. These physiological changes can modify ARV exposure, rendering it challenging to predict pharmacokinetic outcomes in pregnant women. Consequently, dosing recommendations and contraindications for ARVs are typically based on studies excluding pregnant women, potentially leading to underestimations of DDI significance in this population [14]. Also, individuals with HIV often have additional comorbidities such as tuberculosis, epilepsy, and other conditions [9,15]. In particular, HIV infection combined with epilepsy or bipolar disorder requires careful management. Current guidelines recommend caution when combining antiretrovirals with anticonvulsants to avoid adverse interactions and ensure effective treatment [16]. Individuals with epilepsy, particularly older adults and those with HIV, have a higher prevalence of comorbid conditions due to both the pathogenic causes of epilepsy and the toxicities of antiepileptic drugs, with HIV-infected patients being especially prone to seizures from various mechanisms and experiencing premature aging-related comorbidities [17,18,19]. The guidelines from the American Academy of Neurology (AAN) and the International League Against Epilepsy (ILAE) focus on selecting antiepileptic drugs (AEDs) for individuals with HIV/AIDS, addressing drug interactions with antiretroviral agents (ARVs). Up to 55% of HIV/AIDS patients on ARVs may need AEDs, as seizure disorders are common [16]. Specific interactions include adjustments in lopinavir/ritonavir dosage with phenytoin [20], zidovudine with valproic acid [21], and lamotrigine (LTG) with ritonavir/atazanavir [22]. The guidelines recommend avoiding enzyme-inducing AEDs with protease inhibitors (PIs) or non-nucleoside reverse transcriptase inhibitors (NNRTIs) to prevent reduced ARV efficacy [23,24]. Monitoring through pharmacokinetic assessments is crucial.

Lamotrigine (LTG) and efavirenz (EFV), commonly used for the treatment of epilepsy and HIV, respectively, can suffer altered pharmacokinetics due to increased estrogen levels and genetic polymorphisms, necessitating careful consideration of dose adjustments [25,26]. LTG levels can drop significantly during pregnancy, primarily due to enhanced clearance mediated by increased glucuronidation [27,28]. Several studies have demonstrated that this reduction, particularly during the second and third trimesters, often requires regular monitoring and dosage adjustments to maintain therapeutic levels [29]. The rapid return of LTG levels to pre-pregnancy concentrations further underscores the need for dynamic and individualized dosing strategies [28,30]. Efavirenz (EFV) is a widely used non-nucleoside reverse transcriptase inhibitor for HIV treatment, particularly in low- and middle-income countries. While historically, there were concerns about its teratogenic potential based on animal studies and isolated case reports of neural tube defects, more recent data have alleviated these concerns. Large-scale studies and meta-analyses have demonstrated that the risk of major birth defects with EFV exposure during pregnancy is not significantly higher than in the general population. For instance, a systematic review found no increased risk of birth abnormalities with EFV exposure during the first trimester, and the overall incidence of birth defects was comparable to the general population [5]. Genetic polymorphisms influence the pharmacokinetics of EFV during pregnancy in CYP2B6, the enzyme responsible for its metabolism. These polymorphisms can significantly affect drug levels, complicating management during pregnancy. Despite this complexity, there is no consensus on the need for routine dose adjustments of EFV during pregnancy [31,32], highlighting the need for further research and careful therapeutic monitoring in this population [26,33].

Further research and in silico studies are needed to understand better the pharmacokinetics and interactions of these drugs during pregnancy, ensuring safe and effective treatment for pregnant women with epilepsy and HIV. Drug-drug interaction studies indicate that EFV induces UGT1A4 and, therefore, could decrease LTG exposure if given combined. The metabolism of LTG is facilitated by UDP-glucuronosyltransferases, primarily UGT2B7, with transporters SLC22A1 and ABCB1 also playing significant roles. Genetic variations in UGT1A4, UGT2B7, ABCB1, and SLC22A1 can affect the production and activity of these proteins [34]. Evidence shows that UGT1A4 is up-regulated by 17β-estradiol (E2) through ERα and Sp1, suggesting that elevated E2 levels during pregnancy increase hepatic UGT1A4 expression, altering the metabolism of its substrate drugs [35]. The CYP primarily metabolizes EFV. However, the glucuronidation of EFV metabolites, such as 8-hydroxyEFV (8-OHEFV) and 8,14-dihydroxyEFV (8,14-diOHEFV), is also carried out by multiple UGT isoforms, including UGT1A1, UGT1A3, UGT1A7, UGT1A8, UGT1A9, UGT1A10, and UGT2B7 [36]. In vitro studies have shown that EFV can be directly glucuronidase to EFV-N-glucuronide by UGT2B7, although this is a minor pathway following the first dose of EFV [37]. While the production of 7-hydroxy-EFV-glucuronide, 8-hydroxy-EFV-glucuronide, and 8,14-dihydroxy-EFV-glucuronide is more consistent, the generation rate of EFV-N-glucuronide varies greatly throughout human micro-some samples [36]. EFV can potentially inhibit the glucuronidation of drugs catalyzed by UGT1A4 and/or UGT1A9. Therefore, potential pharmacokinetic drug interactions due to the inhibition of UGT1A4 and UGT1A9 should be examined in vivo to assess the clinical relevance of the inhibitory interaction of EFV with UGT1A4- and UGT1A9-substrate drugs [38]. LTG has been found to significantly benefit patients with NRTI-induced neuropathy, a condition that can occur in patients taking certain antiretroviral medications [39]. The antiretroviral drug most frequently linked to central nervous system toxicity, resulting in sleeplessness, agitation, and vivid nightmares, is the NNRTI. Recent research indicates that individuals with different cytochrome P450 2B6 alleles may be more susceptible to experiencing these negative effects [40,41,42].

Both LTG and EFV fall into the category of narrow therapeutic index (NTI) drugs [43,44]. The therapeutic range for LTG is relatively small, and small changes in drug levels can lead to significant changes in therapeutic effects and risk of adverse effects. Due to its narrow therapeutic index and the potential for severe side effects, if levels become too high or too low, Therapeutic Drug Monitoring (TDM) is often recommended for LTG [45]. The EFV therapeutic window is relatively narrow, and variations in plasma levels can significantly impact both efficacy and toxicity. TDM is less commonly performed for EFV than LTG, but it is still important in certain clinical situations [43,46,47]. Using a virtual approach instead of standard TDM for adjusting anticonvulsant and antiviral drug dosages during pregnancy can offer several significant advantages. A virtual approach can utilize physiologically based pharmacokinetic (PBPK) models to simulate how drugs are metabolized in pregnant women, considering individual variations in physiology and biochemistry. These models can predict optimal drug dosages based on a patient’s specific characteristics (e.g., age, weight, stage of pregnancy), leading to more tailored and potentially more effective treatment plans [48,49,50,51]. Unlike standard TDM, which requires blood samples at specific intervals, virtual approaches can continuously monitor drug levels and predict the need for dosage adjustments without invasive procedures. Changes in drug metabolism due to physiological changes in pregnancy can be accounted for in real time, providing immediate recommendations for dosage adjustments. This minimizes the need for frequent blood tests and lab analyses. Virtual approaches can be more cost-effective in the long run. Virtual models can dynamically adjust to the rapid physiological changes that occur during pregnancy, such as increased blood volume and altered enzyme activity, ensuring drug dosages remain appropriate throughout the different stages of pregnancy. These approaches can lead to better health outcomes for both the mother and the fetus, making them a promising complement to traditional methods.

**Figure 1:**
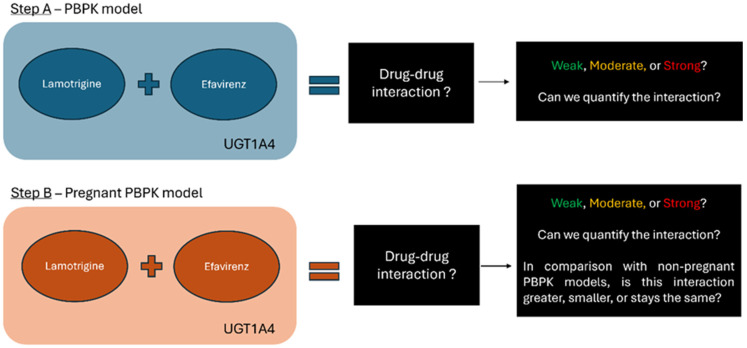
Schematic Overview of the Research Objective: The study aims to model the physiological pharmacokinetics of LTG and EFV in both pregnant and non-pregnant individuals. It seeks to determine the potential for drug-drug interaction (DDI) between these two drugs, focusing on UGT-mediated metabolism. Both LTG and EFV are metabolized by UGT1A4, making this pathway a potential interaction across different physiological states.

Moreover, given the scarcity of clinical data on DDIs in pregnant women, particularly involving UGT substrate drugs like LTG and EFV, in silico modeling offers a valuable approach to predicting pharmacokinetic changes and potential interactions. This study aimed to model the pharmacokinetics of LTG and EFV in pregnant women using a physiologically based approach to understand better and manage these interactions (Figure 1) and to understand the difference in comparison with non-pregnant individuals. In silico research can bridge the gap in clinical data by simulating drug metabolism and interaction scenarios, helping to predict changes in drug exposure and efficacy during pregnancy. Understanding these dynamics is crucial for ensuring the safe and effective use of medications in pregnant women living with HIV and other conditions requiring anticonvulsant therapy. This provides insights into the pharmacokinetic profiles of LTG and EFV in pregnant women, contributing to improved clinical management and patient outcomes. By elucidating these dynamics, PBPK models can inform clinical decision-making, ensuring that pregnant women receive safe and effective treatment for epilepsy and HIV. This approach aims to enhance maternal and fetal health outcomes, balancing the mother’s therapeutic needs with the developing fetus’s safety.

## 2. Materials and Methods

### 2.1. Clinical PK Data Collection and Enzyme Information

We collected relevant pharmacokinetic (PK) data from clinical studies using PubMed, focusing on pregnant and non-pregnant populations treated with LTG and EFV. The data included key metrics such as plasma concentration profiles, area under the curve (AUC), maximum concentration (Cmax), and clearance (CL). We used the WebPlotDigitizer tool (https://automeris.io/wpd/?v=4_8), version 4.8, accessed on 12 January 2024, from Bangalore, India) to extract and quantify these data points. This tool enabled us to digitize and analyze time versus plasma concentration data from graphical representations in the studies, which is crucial for developing accurate pharmacokinetic models and assessing potential drug-drug interactions.

### 2.2. Model Development for Non-Pregnant Individuals

All models were created using GastroPlus v.9.8.3 (Simulation Plus Inc., Lancaster, CA, USA). For LTG, PBPK models were replicated based on existing pharmacokinetic data and established methodologies, especially Conner et al. [52] and Caleffi-Marchesini et al. [53]. The input of physicochemical, PK, and physiological parameters for the LTG PBPK model is detailed in Table 1. The digitized values from the selected articles (Table S1) were then loaded into GastroPlus, where we utilized the PKPlus model to develop compartmental models and estimate parameters such as clearance (CL/F) and volume of distribution (Vd). Subsequently, we constructed PBPK models tailored to the demographic characteristics outlined in the studies from which we gathered data. The PBPK physiologies, including organ weights, volumes, and blood flows, were generated using the Population Estimates for Age-Related Physiology (PEAR Physiology™, which is part of GastroPlus software), and the Lukacova (Rodger-single) method was employed as a perfusion-limited model to calculate the tissue-to-plasma partition coefficient. This development incorporated absorption inputs such as in vitro solubility in relevant media, effective permeability (Peff), formulation properties, and physiological factors affecting first-pass metabolism, gastric emptying, intestinal transit time, and transport. We utilized the software’s default IR tablet and the Johnson dissolution model. We used the Caleffi-Marchesini et al. sensitivity analysis (PSA), which highlighted the impact of solubility and physiological factors on pharmacokinetic (PK) parameters—adjustments to stomach transit time improved model predictivity. A stomach transit time of 0.5 h provided the best results [53,54]. The Vmax and Km values for non-pregnant individuals were collected from the literature and inputted in the enzyme table.

Compound parameters from the GastroPlus software, v.9.8.3. library were utilized to develop the Efavirenz (EFV) model, leveraging an existing EFV PBPK model available within GastroPlus [55]. Plasma concentration versus time (Cp-time) profiles digitized from literature sources were used for model building (Table S2). The PBPK physiologies, including organ weights, volumes, and blood flows, were generated using the Population Estimates for Age-Related Physiology (PEAR Physiology™). Efavirenz tissue distribution was modeled using a perfusion-limited approach for all tissues, with tissue-to-plasma partition coefficients (Kps) predicted by the default Lukacova model. Efavirenz absorption from the gut was modeled as a passive diffusion process. Metabolism was primarily attributed to CYP2B6 and CYP3A4 isozymes, with additional consideration given to UGT1A9 and UGT1A4 for modeling purposes of drug-drug interactions. The Vmax and Km values for CYP2B6 and CYP3A4 were preloaded in the existing EFV PBPK model on GastroPlus. We assumed the values for this CYP were representing intermediate metabolizers. Since genetic polymorphisms influence EFV metabolism, it is crucial to identify the type of metabolizer being represented in the PBPK model.

**Table 1:** Input parameters assumed in LTG and EFV PBPK model.

Parameters	LTG	EFV	Reference LTG and EFV
**Physicochemical and Blood Binding**			
MW (g/mol)	256.09	315.68	[52,56,57]
log P	1.70	4.6	[55,58]
pK_a_ (Base)	4.41	10.2	Predicted with Henderson-Hasselbalch equation using GastroPlus™
Solubility factor	12.09	4342.8	Predicted using GastroPlus™
Solubility (mg/mL)	2.54 (pH 1.2); 0.38 (pH 4.5); 0.24 (pH 6.8); 0.25 (pH 7.4); 0.21 (pH 8.0)	9.0 × 10^−3^ (pH = 6.9641)	[54]/Predicted using GastroPlus™
B:P	1.00	0.74	[55,59]
Fup	0.45	0.22	[55,60]
**Absorption**			
Dosage form	IR	IR:Capsule	
P_eff_ (cm/s)	7.76 × 10^4^	1.07 × 10^4^	[52,55]
Diffusion coefficient (cm^2^/s)	0.84 × 10^−5^	0.84 × 10^−5^	Predicted using GastroPlus™
Particle size distribution	Log-normal	Log-normal	Default GastroPlus™
Mean particle radius (µm)	25.00	5	Default GastroPlus™
Particle density (g/mL)	1.20	1.20	Default GastroPlus™
Dose volume (mL)	250	250	Default GastroPlus™
Precipitation model	First order	First order	Default GastroPlus™
Precipitation time (s)	900	900	Default GastroPlus™
Solubility FaSSGF (mg/mL)	3.48	6.5	[54]/Predicted using GastroPlus™
Solubility FaSSIF (mg/mL)	0.35	5	[54]/Predicted using GastroPlus™

MW (g/mol): Molecular weight of the compound; log P: Partition coefficient; pK_a_: The dissociation constant, indicating the strength of the compound as a base; B:P: Blood to plasma concentration ratio; Fup: Fraction unbound; P_eff_ (cm/s): Effective permeability coefficient.

### 2.3. PBPK Model Development for Pregnant Individuals

Following the validation of models for non-pregnant individuals, we transitioned to the pregnant PBPK (p-PBPK) models to simulate PK in pregnant individuals. Separate p-PBPK models were created for each drug (LTG and EFV) using the Population Estimates for Age-Related Physiology (PEAR) module. These calculations account for the current body weight (pregnancy weight plus weight gain) at various gestational ages, following the equations used for healthy subjects. The expression levels of transporters (both influx and efflux) were automatically scaled according to organ volume. These models account for variations in tissue sizes, blood flow rates, enzyme expression levels, plasma protein binding, and other physiological factors that influence drug PK in both the pregnant individual and the fetus.

Vmax and Km values were not manually adjusted to reflect the changes in metabolic enzyme activity caused by hormonal and physiological shifts during pregnancy. Instead, the Population Estimates for Age-Related Physiology (PEAR) module was used, and when pregnancy was selected as the PK physiology, Vmax and Km values from the non-pregnant population were recalculated to fit the model. This adjustment was validated by comparing it with values from the available literature [32,34]. During pregnancy, the relative activity of UGT1A4 and UGT1A3 in LTG metabolism increases. LTG is primarily metabolized by UGT1A4, with a minor contribution from UGT1A3. For EFV, metabolism is primarily mediated by cytochrome P450 (CYP) enzymes, particularly CYP2B6, with a lesser role for CYP3A4, but a fraction of EFV is metabolized by UGT enzymes [38]. Therefore, the accuracy of these metabolic differences may only be somewhat reliable due to the absence of in vitro experiments to confirm the exact values.

### 2.4. Model Evaluation and Validation

The evaluation of the model involved two main steps: first, calculating the ratio between observed and predicted values for the area under AUC0–t and Cmax. Second, we visually inspected the predicted plasma profile with the clinical data obtained from the literature (Figures S1 and S2). This comparison overlapped simulation predictions with data from studies involving healthy, non-pregnant individuals for LTG and EFV. Conditions from these clinical studies were replicated in the simulations, and the results were compared with observed data. The model was considered acceptable when the percentage of prediction error (%PE) for AUC0–t and Cmax was less than 25%. We have replicated pharmacokinetic parameters for single doses of LTG at 25, 75, 100, or 200 mg (Table S1) and EFV doses of 400 and 600 mg, administered orally as a single dose (Table S2).

Statistical metrics were calculated, including the average fold error (AFE) and the average absolute fold error (AAFE), the Mean Absolute Error (MEA), and the Root Mean Square Error (RMSE). The AFE provides insight into the model’s degree of inaccuracy and potential prediction error. AAFE offers a measure of prediction precision, reflecting how closely the model’s predictions align with observed values. MAE indicates the average magnitude of prediction errors. At the same time, RMSE emphasizes the effect of larger deviations between predicted and observed values, thereby enhancing the overall robustness of the model-validation process [61,62].

To validate the p-PBPK models for LTG and EFV, we utilized clinical data from studies involving pregnant individuals, as summarized in Tables S1 and S2. The conditions described in these studies were simulated, and the extracted data was compared against predictions from the simulations whenever possible. The accuracy and precision of the model predictions were also assessed by comparing observed and predicted pharmacokinetic parameters, specifically AUC and Cmax. However, finding studies that provided detailed concentration-time (Cp) profiles or pharmacokinetic parameters across different gestational weeks was challenging for both drugs. Although more data were available for EFV, these were primarily limited to the third trimester. In contrast, most LTG studies focused on changes in drug clearance related to increased enzyme activity during pregnancy.

### 2.5. Dose Simulation

The dosing of lamotrigine during pregnancy is complex and varies by trimester due to changes in metabolism and drug clearance. To address this, we simulated a constant 200 mg dose throughout pregnancy and adjusted doses for different gestational ages (GA) to better understand the pharmacokinetics of lamotrigine at various stages. The simulation of the 200 mg dose allowed us to evaluate how well the model describes lamotrigine’s pharmacokinetics across different trimesters. Additionally, adjusting the doses provided more realistic values reflective of physiological changes. The rationale behind the dose decision is outlined in Table 2.

For efavirenz (EFV), the standard dosing during pregnancy is 600 mg daily. Reduced doses, such as 400 mg daily, may be insufficient due to the induction of CYP2B6 during pregnancy [38,63,64,65,66]. Based on this rationale, we move forward to the DDI simulations.

### 2.6. DDI Module

Using the DDI module of GastroPlus, we performed steady-state predictions and dynamic simulations to qualitatively and quantitatively analyze LTG and EFV interactions. We defined EFV as the perpetrator drug and LTG as the victim. The interaction was simulated for the intake of 100 to 400 mg LTG and 600 mg EFV using both PBPK and pregnant-PBPK models and the intake of 200 mg LTG and 400 mg of EFV using the Adult standard PBPK model. For the pregnant models, we simulated drug-drug interactions at 10, 20, 30, and 40 weeks of gestation. As stated in Table 2, we attributed a specific dose to each gestational week.

Table 3 and Table 4 provide crucial information for our drug-drug interaction (DDI) simulation. Table 3 details the values for LTG. Although Vmax and Km values are not explicitly listed for pregnant states in our data, the variations in Clint values—calculated as Clint = Vmax/Km—suggest that these underlying parameters are adjusted in the model to reflect observed changes in enzyme activity, as we have mentioned above. Table 4 provides enzyme values for EFV used in our DDI predictions, focusing on its inhibitory effects as the perpetrator. It is important to note that these calculations were not derived from in vitro experiments. Instead, Vmax and Km values were based on the research by Ji et al. [38]. Unfortunately, we could not obtain these values specifically for pregnant populations. Nevertheless, the software adjusted these values to account for physiological changes during pregnancy [13,67].

## 3. Results

### 3.1. Adult PBPK and Pregnancy-PBPK Models for Lamotrigine

The model shows varying accuracy in predicting lamotrigine pharmacokinetic parameters (Figure S1 and Table 5). The parameters evaluated include the fraction absorbed (F%), Cmax, time to peak concentration (Tmax), area under the concentration-time curve over 24 h (AUC 0–24), area under the concentration-time curve extrapolated to infinity (AUC 0-inf), and maximum liver concentration (Cmax Liver). For the adult PBPK model, the calculated volume of distribution (Vd) was 1.13 L/kg, which is in agreement with the values (0.9–1.5 L/kg) published in the literature [60,68]. The estimated total clearance, comprising renal excretion and metabolic clearance, was 2.09 L/h, consistent with values reported in the literature ranging from 1.3 to 2.51 L/h [69]. For the p-PBPK model, simulations were performed using clinical data from all trimesters of pregnancy. Clearance data were available and compared across all three trimesters [30]. However, assessments of AUC and Cmax were limited to the eighth month of pregnancy and baseline measurements.

In Table 5, the predicted AUC values are generally close to the observed values, with percentage prediction errors (%PE) ranging from −14.35% to 10.92%. This indicates that the model’s prediction of overall drug exposure over time is fairly accurate. The predicted Cmax values show a higher degree of variability, with %PE ranging from −3.13% to 20.89%. This suggests that while the model predicts peak plasma concentrations reasonably well, there are some deviations, particularly at lower doses. Nonetheless, %PE was less than 25%, which is an acceptable range. For the p-PBPK model, in Table 6, the predicted AUC in pregnant individuals is close to the observed values, with a %PE of 9.84%. This indicates that the model has a good ability to predict the overall exposure of lamotrigine in the body after oral administration, suggesting that the model accurately captures the drug’s pharmacokinetics in terms of overall exposure. The predicted Cmax of 5.05 µg/mL is very close to the observed value of 5.3 µg/mL, with a small percentage error of −4.064%. This suggests that the model is quite accurate in predicting the peak concentration of lamotrigine following the 400 mg dose. Such a small deviation is within acceptable limits, indicating the model’s reliability in forecasting the peak levels of the drug. All models were deemed acceptable if the percentage of prediction error (%PE) for AUC0–t and Cmax was less than 25%.

#### 3.1.1. LTG’s Model Evaluation Metrics

Based on the statistical metrics, these results provide a statistical evaluation of the pharmacokinetic model’s predictive performance for both non-pregnant and pregnancy conditions. All AFE values are close to 1, which generally indicates good predictive accuracy for both Cmax and AUC in standard and pregnancy conditions. AAFE values close to 1 imply good predictive accuracy. The values here suggest the model’s predictions are generally reliable, though there’s a slightly larger error margin for AUC in the standard condition. We assumed that an AFE close to 1 indicates strong agreement between predicted and observed values, with a typical acceptable range being 0.8 to 1.25. An AAFE near 1 reflects good predictive accuracy, while an AAFE less than 2 was generally deemed acceptable. Values significantly above 2 suggested poor predictive accuracy [62,74].

In Table 7, the AFE values are close to 1 for both standard and pregnancy conditions, indicating that the model’s predictions for both Cmax and AUC are generally accurate. AAFE values close to 1 across conditions reflect good precision. AAFE values are within acceptable ranges, showing that the model’s predictions are generally precise. For standard conditions, the MAE and RMSE for AUC are notably high, indicating substantial errors in predicting total drug exposure. In pregnancy conditions, the MAE and RMSE for AUC are lower but still notable.

The model is suitable for use, given its generally good predictive accuracy and precision for both Cmax and AUC in standard and pregnancy conditions. Regular updates or refinements based on new data could help improve its performance further.

#### 3.1.2. LTG’s p-PBPK Model Reflects the Pharmacokinetics Changes Influenced by Pregnancy with the Decrease of Plasma Concentrations and Overall Exposure as Gestational Age Advances

Table 8 presents the simulation results for LTG administered at a constant dose of 200 mg across various gestational ages, also comparing with the non-pregnant female, providing insights into how PK parameters such as Cmax, Tmax, AUC, and bioavailability are affected by pregnancy and gestational age. The Fa (%) ranges from 99.745% to 99.761% across different conditions, indicating that the fraction of the administered dose that reaches systemic circulation remains relatively constant.

The peak plasma concentration decreases from 2.88 µg/mL in the first trimester to 2.41 µg/mL in the fourth trimester. This trend suggests a reduction in plasma concentration as pregnancy progresses, which aligns with known physiological changes during pregnancy, such as increased blood volume and altered drug metabolism [8,13]. The time to reach peak concentration slightly increases from 0.8 h in the first trimester to 1.04 h in the fourth trimester. This gradual increase in Tmax reflects delayed absorption or increased gastric transit time associated with later stages of pregnancy.

The area under the curve (AUC 0-inf), which represents the total drug exposure over time, remains relatively stable across gestational ages, with minor fluctuations. This stability indicates that despite changes in peak concentration and absorption time, overall drug exposure does not vary drastically. The AUC from time zero to the last measurable concentration shows a slight decrease from 44.92 µg-h/mL in the first trimester to 38.454 µg-h/mL in the fourth trimester. This decline in AUC over the course of pregnancy suggests reduced drug exposure with advancing gestational age. The liver concentration of LTG remains relatively stable across different gestational ages, indicating consistent hepatic exposure to the drug despite changes in systemic plasma levels.

**Figure 2:**
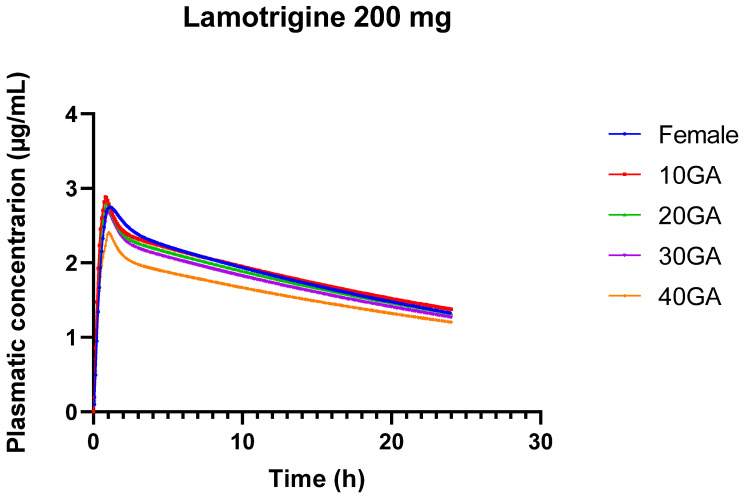
24-h plasma concentration profiles of 200 mg LTG in healthy female individuals (blue) and for different gestational weeks: 10 (red), 20 (green), 30 (purple), and 40 (orange).

The decrease in Cmax and the slight reduction in AUC with advancing gestational age suggest that LTG levels in the plasma are lower in the later stages of pregnancy. This reduction can be attributed to physiological changes such as increased blood volume, enhanced renal clearance, and changes in liver enzyme activity, which can all influence drug metabolism and clearance. The gradual increase in Tmax indicates that the time required to reach peak LTG concentration is slightly extended as pregnancy progresses. This could be due to changes in gastric emptying or other absorption-related factors. Although there are fluctuations, the overall stability of AUC values suggests that the total drug exposure over time remains consistent. This stability is crucial for maintaining therapeutic efficacy while accounting for the physiological changes that occur during pregnancy. The model helps understand how LTG levels might change throughout pregnancy, providing a basis for adjusting dosing to maintain therapeutic levels. Figure 2 serves to visualize the Cp vs. time profiles for the different conditions.

The pharmacokinetics of LTG during pregnancy can undergo significant alterations, resulting in substantial drops in drug concentrations. This is primarily due to the physiological and hormonal changes that occur during pregnancy, including increased metabolism and altered drug clearance. During pregnancy, LTG clearance increases noticeably and more so than with previous AEDs. The results from the 24-h simulation of LTG across different doses and gestational ages reveal several important trends and implications for both drug pharmacokinetics and potential clinical use (Table 9). The results show minor differences in LTG pharmacokinetics between genders, with slightly increased AUC and Cmax in females compared to the standard condition. This suggests that gender may influence drug absorption and exposure, but the differences are relatively small.

The comparison between the Female group and 10 GA reveals a notable increase in drug exposure at early pregnancy stages (10 GA). The higher Cmax and AUC at 10 GA indicate that LTG levels are higher during early pregnancy, possibly due to altered absorption or changes in metabolism and clearance. The model demonstrates that the increased AUC and Cmax at early gestational ages suggest that dosing strategies may need to account for changes in drug exposure during pregnancy. Higher doses or more frequent monitoring might be necessary to maintain therapeutic efficacy as pregnancy progresses.

On the other hand, simulating a constant dose across different populations and varying doses across gestational ages provides a comprehensive understanding of how pharmacokinetics are influenced by physiological changes (Table 9). This approach enhances the ability to tailor dosing strategies, improve therapeutic outcomes, and ensure safety across diverse patient groups and stages of pregnancy.

### 3.2. Adult PBPK and Pregnancy-PBPK Models for EFV

The results in Table 10 present a comparison between predicted (Pred) and observed (Obs) pharmacokinetic (PK) parameters for EFV at two different doses (400 mg and 600 mg) as simulated using a PBPK and clinical studies. For the adult PBPK model the Vd of EFV can vary based on an individual’s metabolic rate, particularly influenced by genetic polymorphisms [31]. Intermediate metabolizers generally have a Vd that falls between the ranges observed in poor metabolizers and extensive (or rapid) metabolizers. Their Vd values are likely to be within this range but may be slightly adjusted based on their specific metabolic capacity [75,76]. The calculated Vd was 1.341 L/kg, and the estimated total clearance was 3.496 L/h. This is consistent with values published in the literature [63].

For the 400 mg dose, the predicted AUC (45.58 µg/mL-h) is lower than the observed AUC (52.86 µg/mL-h), with a percentage error (%PE) of −13.78%. This suggests that the model underpredicts the drug exposure at this dose. The predicted Cmax (3.24 µg/mL) is slightly higher than the observed value (2.98 µg/mL), resulting in a %PE of 8.89%, which is acceptable. For 600 mg the predicted AUC (68.99 µg/mL-h) is substantially lower than the observed AUC (100.61 µg/mL-h), with a %PE of −31.42%. This indicates an underprediction of drug exposure at this higher dose. The predicted Cmax (4.9 µg/mL) is lower than the observed value (6.034 µg/mL), resulting in a %PE of −18.79%, which is acceptable. In this particular case, the model was deemed acceptable even though the %PE for AUC 0–t was more than 25% because the %PE in the table compares the predicted values from the model with the observed values calculated using the digitized data from the original studies (Villani et al. [77]). Although the model didn’t perfectly fit the observed data in Villani et al.’s study (as reflected in the %PE values), it’s important to note that the simulated values for Cmax (4.9 µg/mL) and AUC (68.99 µg/mL-h) are still within the reported ranges in Villani et al.’s article (Table S2). Specifically, Cmax reported 4.0 ± 1.7 µg/mL, which means a range of 2.3 to 5.7 µg/mL, and AUC reported 57.15 ± 27.3 µg/mL-h, which translates to a range of 29.85 to 84.45 µg/mL-h. The model’s outputs (Cmax = 4.9 µg/mL and AUC = 68.99 µg/mL-h) fall within these ranges, indicating that despite the apparent discrepancies (%PE), the model’s predictions are reasonably consistent with the variability observed in the actual study. Given that the model’s predictions fall within the expected range of values reported by Villani et al. [77] and considering that the model accurately fits the data from Cerrone et al. [78] (as shown by the smaller %PE for the 400 mg dose), it was deemed appropriate to continue using this model for further investigations.

**Table 10:** Model validation results: pharmacokinetic parameters predicted (Pred) and observed (Obs) for oral administration of efavirenz in adults.

Dose	AUC 0–t (µg/mL-h)			Cmax (µg/mL)			Tmax		
	Pred	Obs	%PE	Pred	Obs	%PE	Pred	Obs	%PE
400 mg Cerrone et al. [78]	45.58	52.86	−13.78	3.24	2.98	8.89	-	-	-
600 mg Villani et al. [77]	68.99	100.61	−31.42	4.9	6.034	−18.79	1.92	3.034	−36.72

Table 11 provides the results of a model validation comparing predicted (Pred) and observed (Obs) pharmacokinetic (PK) parameters for oral administration of EFV during pregnancy at two different gestational ages (GA): 33 weeks and 23 weeks. The model’s accuracy is evaluated by the percentage error (%PE) between the predicted and observed values. For the 33 Weeks Gestation (Cressey et al. [26]) the model predicted an AUC of 20.77 µg/mL-h, while the observed AUC was 165.18 µg/mL-h, resulting in a percentage error (%PE) of −87.43%. However, it’s important to note that if we compare the simulated AUC value with the range reported in Cressey et al.’s study, the predicted value still falls within this range (Table S2: AUC reported range 13.5 to 220.3 µg/mL-h). This indicates that despite the large %PE, the simulated AUC is not entirely out of bounds when considering the variability in the study population. The predicted Cmax was 2.79 µg/mL compared to the observed Cmax of 8.33 µg/mL, resulting in a %PE of −66.39%. Again, while there is a large discrepancy, the predicted Cmax might still be within the range of values reported in the study (Table S2: Cmax reported range 1.90 to 12.22 µg/mL), highlighting that the model’s predictions are not entirely inaccurate but do reflect the high variability typical in pregnant populations.

On the other hand, for the simulation of 23 Weeks Gestation (Lartey et al. [33]), the predicted AUC was 22.27 µg/mL-h while the observed AUC (from the digitization of the cp vs. time profile) was 43 µg/mL-h, with a %PE of −48.22%. However, in this case, the underprediction does not fall within the range of values reported by Lartey et al. in their study (Table S2: AUC reported range is from 39.07 to 70.6 µg/mL-h). This suggests that while the model underestimates the AUC, it does so within an acceptable margin considering the reported variability. The predicted Cmax of 2.99 µg/mL closely matched the observed Cmax of 3.09 µg/mL, with a very small %PE of −3.22%. This indicates that the model performed well in predicting the peak concentration at 23 weeks’ gestation, with the predicted value falling well within the expected range.

For both the 33-week and 23-week gestational ages, we observe a significant underprediction of AUC by the model at the 600 mg dose, which is consistent with the earlier observations. The large negative %PE values indicate that the model may not accurately represent the total drug exposure during pregnancy, especially at higher doses. While the model does show significant discrepancies, particularly in AUC predictions for the 600 mg dose, we considered it could still be used for DDI studies with LTG, given that the primary focus of such studies is on the interaction mechanism rather than precise PK values.

#### 3.2.1. Model Validation

Table 12 presents the statistical metrics for the evaluation of the EFV PBPK model, comparing predictions for standard (adult male) and pregnant populations. The model’s performance for standard (adult male) conditions is generally acceptable, with AFE and AAFE values within the acceptable range, though there is still some moderate deviation. For the p-PBPK model, we can see significant challenges in accurately predicting EFV pharmacokinetics during pregnancy. The AFE and AAFE values are well below the acceptable thresholds, indicating that the model underestimates both Cmax and AUC significantly. The high MAE and RMSE values further underscore the difficulties in achieving accurate predictions for pregnant individuals.

For non-pregnant individuals, the model can be used with reasonable confidence for predicting EFV pharmacokinetics. The p-PBPK model has poor performance in predicting EFV pharmacokinetics during pregnancy, suggesting that it may not be reliable for clinical use in pregnant individuals. The model should be refined to better account for pregnancy-specific physiological changes and genetic variability, which significantly impact drug metabolism and pharmacokinetics.

#### 3.2.2. Challenges in EFV Modeling: Impact of Genetic Polymorphisms, Gender Differences, and Dose Variations

Table 13 illustrates dose-dependent changes and gender differences in EFV pharmacokinetics. In the simulation using the standard PBPK model, Cmax increases with dose for both 400 mg and 600 mg, reflecting the expected pharmacokinetic behavior where higher doses result in higher peak concentrations. The AUC also increases with dose, consistent with the expected increase in overall drug exposure. The AUC 0-inf values are significantly higher in males compared to females, indicating greater overall exposure in males. In the female PBPK model, Cmax also increases with dose, but the values remain consistently lower in females compared to males for both doses. The AUC is lower in females than in males at both doses, suggesting reduced overall exposure in females. This could be attributed to differences in metabolism or distribution. AUC 0-inf values are also lower in females for both doses, reflecting a reduced extent of drug exposure.

**Figure 3:**
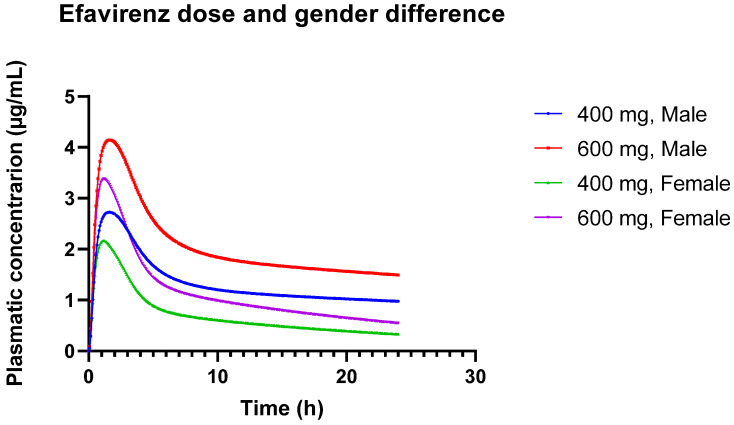
Plasmatic concentration profiles of Efavirenz for different doses (600 mg and 400 mg) in different genders (male and female).

These observed differences in pharmacokinetics between males and females may be due to physiological and metabolic differences. Females may metabolize or eliminate the drug differently than males, which could impact overall drug exposure and peak concentrations. These results underscore the importance of considering gender differences in pharmacokinetic modeling and drug dosing. The lower Cmax and AUC in females suggest that dosing adjustments might be necessary to ensure therapeutic efficacy and avoid underdosing. Figure 3 provides a visual representation of the different concentration versus time profiles.

The results from the simulation of 600 mg of EFV over 24 h, using both the female and pregnant PBPK models, provide insights into the pharmacokinetics of EFV across different gestational ages. The bioavailability of EFV decreases slightly as gestational age advances, from 85.63% in non-pregnant females to 79.67% at 40 weeks of gestation. This trend suggests that the drug’s absorption or systemic availability is somewhat reduced in the later stages of pregnancy.

In Table 14, there is a gradual decline in Cmax from 3.38 µg/mL in non-pregnant females to 2.58 µg/mL at 40 weeks’ gestation. This decrease indicates that the maximum concentration of EFV in the bloodstream decreases as pregnancy progresses. The AUC0–24 decreases from 27.92 µg-h/mL in non-pregnant females to 19.85 µg-h/mL at 40 weeks’ gestation. This reduction reflects lower overall drug exposure over the 24-h period in later stages of pregnancy. Similarly, AUC0-inf decreases from 40.87 µg-h/mL in non-pregnant females to 27.53 µg-h/mL at 40 weeks’ gestation. This further confirms the reduced overall drug exposure throughout the entire dosing interval during pregnancy. The liver concentration of EFV remains relatively stable across different gestational ages, ranging from 17.22 to 17.96 µg/mL. This stability suggests that hepatic concentrations of EFV are less influenced by gestational age compared to plasma concentrations.

**Figure 4:**
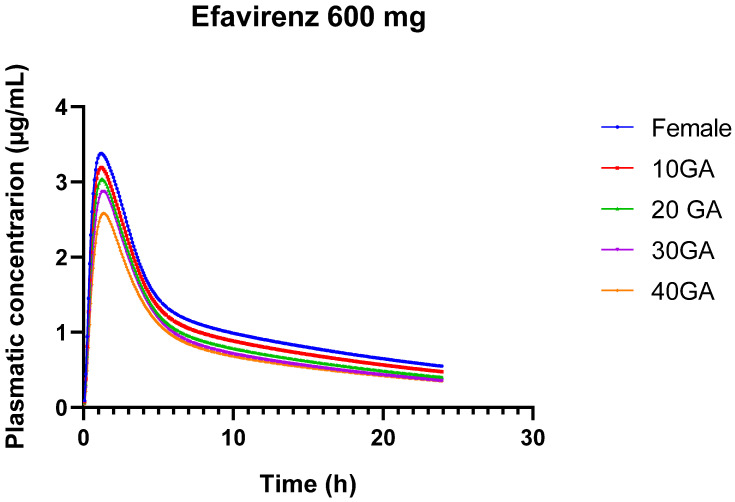
24-h plasma concentration profiles of 600 mg EFV in healthy female individuals (blue) and for different gestational weeks: 10 (red), 20 (green), 30 (purple), and 40 (orange).

While the simulations provide valuable insights into EFV pharmacokinetics across different gestational ages, the model’s current limitations in predicting AUC and precision highlight the need for further development and validation to ensure its clinical utility for pregnant individuals. Figure 4 provides a visual representation of the different concentration versus time profiles for the different conditions.

### 3.3. DDI Module: Dynamic Simulation of LTG as the Victim and EFV as the Perpetrator

The results from the dynamic simulations across different GA for LTG and EFV provide insights into how DDI impacts pharmacokinetic parameters. In Table 15 and Table 16, we provide the results that quantify the interaction between LTG and EFV for pregnant and nonpregnant individuals, with results expressed as ratios of the DDI to baseline values (the comparison includes baseline and DDI scenarios for LTG and EFV; for more detailed results, we have the full table on the Supplementary Materials, Table S3).

For LTG, Cmax values during co-administration are nearly identical to those during baseline and pregnancy, showing a minimal increase, indicating that EFV has a negligible effect on LTG’s Cmax. AUC (0-inf) values during co-administration are nearly identical to those during baseline and pregnancy, indicating that EFV has a negligible effect on LTG’s overall exposure. For EFV, the co-administration with LTG, Cmax decreases marginally, which indicates a minor impact of LTG on EFV’s Cmax. AUC (0-inf) values during co-administration show a slight reduction, indicating a minor impact of LTG on EFV’s overall exposure.

For LTG both Cmax and AUC (0-inf) progressively increase during pregnancy, peaking at 30 weeks GA. The co-administration with EFV has a minimal effect on these parameters, suggesting that LTG’s pharmacokinetics are not significantly altered by EFV. In the case of EFV both Cmax and AUC (0-inf) progressively decrease during pregnancy, with the greatest reduction seen at 40 weeks’ GA. The co-administration with LTG leads to a slight additional decrease in these values, but the impact remains minimal.

Overall, the changes in Cmax and AUC (0-inf) during pregnancy indicate that while pregnancy alters the pharmacokinetics of both drugs, the interaction between LTG and EFV is minimal, and the changes are not clinically significant.

We have also conducted a dynamic simulation to evaluate the interaction between LTG and EFV using the standard adult PBPK models for both drugs (see Table 16). Specifically, we simulated the interaction of 200 mg LTG with 400 mg EFV to assess potential differences in their interaction. The model validation for EFV using a 400 mg dose was deemed acceptable, so this simulation aimed to investigate whether any observed differences in the interaction might highlight limitations in the EFV PBPK model, particularly concerning higher doses (600 mg). If significant discrepancies were identified, they could indicate potential inadequacies in the EFV PBPK model.

The results we obtained are similar to what we present in Table 15. The impact of the DDI on LTG is minimal. The co-administration of EFV has a negligible effect on LTG’s Cmax, indicating that LTG’s peak concentration remains virtually unchanged when taken with EFV. The co-administration of EFV leads to a slight increase in LTG’s overall exposure. Although the increase is small, it suggests that EFV may slightly enhance the absorption or reduce the clearance of LTG. For EFV, the co-administration of LTG leads to a very slight decrease in EFV’s Cmax, suggesting a minimal interaction effect on EFV’s peak concentration. The co-administration of LTG leads to a notable decrease in EFV’s overall exposure. This decrease indicates that LTG may slightly induce the metabolism or enhance the clearance of EFV, resulting in reduced drug exposure. The dynamic simulation suggests that the interaction between LTG and EFV results in only minor changes in Cmax for both drugs, with more noticeable changes in AUC (0-inf), particularly for EFV, where the decrease may be clinically relevant.

## 4. Discussion

### 4.1. LTG Model for Pregnant and Non-Pregnant Individuals: Clinical Applicability

Modeling LTG presents significant challenges due to its complex absorption profile, variable bioavailability, and the influence of physiological factors such as gastric transit time, pH levels, and enzyme activity [53,54]. These factors are particularly difficult to predict accurately in PK models, especially during pregnancy, when physiological changes further complicate the drug’s behavior in the body. During pregnancy, changes in blood volume, enzyme activity (particularly UGT1A4, which metabolizes LTG), and renal clearance add further complexity. The fact that our model could reasonably predict AUC and Cmax suggests it adequately incorporates these pregnancy-related changes, even though Tmax remains challenging to predict. The difficulty in obtaining reliable clinical data, especially concentration-time profiles (Cp) across different gestational ages, further complicates model validation. This limitation impacts the precision of our model predictions.

Despite these challenges, our model provides valuable insights into LTG´s pharmacokinetics across different stages of pregnancy. The reasonable accuracy in predicting AUC and Cmax is particularly important for dose adjustments, as these parameters are closely related to therapeutic efficacy and safety. The discrepancies in Tmax prediction, while notable, are less likely to impact clinical decision-making, as Tmax is generally less critical for drugs like LTG, where maintaining consistent exposure is more important than the timing of peak concentration. The model’s success in reasonably predicting AUC and Cmax suggests that it is robust enough to inform clinical dosing strategies, especially in the context of pregnancy where physiological changes significantly impact drug pharmacokinetics. Further refinement of the model and acquisition of more comprehensive clinical data could enhance its predictive accuracy.

Pregnancy induces significant physiological changes, such as increased renal blood flow and altered metabolism, which can affect drug clearance and distribution. The rapid decline in LTG serum concentrations was primarily due to increased renal blood flow, while later changes were influenced by estradiol-induced glucuronidation [29,35]. These dynamic changes over time make it difficult to create a stable model that accurately reflects the pharmacokinetics throughout pregnancy. Our study was conducted exclusively using in silico data, relying on computational models and existing literature to simulate PK parameters. While this approach offers significant advantages, such as the ability to rapidly generate predictions and explore a wide range of scenarios, it also comes with limitations. One key limitation is that we were unable to perform in vitro experiments to directly determine Vmax and Km values for UGT enzymes. As a result, the model adjustments were based solely on available literature data, which may not fully capture the nuances of enzyme activity under various physiological conditions. Additionally, the sample size for certain parameters within the study was relatively small, which could affect the robustness of our statistical analyses and limit the generalizability of our findings. The sample size was constrained by the existing literature, as no new clinical study was initiated to gather more controlled and comprehensive data. Small sample sizes can lead to overfitting in models, where the model may perform well on the data used to create it but may not generalize effectively to a broader population. This can also result in an incomplete representation of variability across different individuals, potentially overlooking rare or outlier responses. The current simulations may not fully capture these dynamics due to limitations in the available data, particularly regarding estrogen conditions and genetic polymorphisms affecting drug metabolism; we can visualize reduced plasma concentrations of LTG. Pregnancy induces numerous physiological changes, such as increased blood volume, enhanced renal clearance, and altered hepatic enzyme activity. These changes can lead to increased drug clearance and reduced plasma concentrations of LTG. Specifically, the activity of enzymes such as UGT1A4 and UGT1A3, which are responsible for the glucuronidation and subsequent clearance of LTG [35,79]. Estrogen has a significant impact on the metabolism of LTG through the induction of UGT enzymes, leading to increased clearance and reduced drug levels. Genetic polymorphisms, particularly those affecting the UGT1A4 and UGT1A3 enzymes, can result in interindividual variability in LTG metabolism [80,81]. These polymorphisms are not fully integrated into the current simulation models, limiting the accuracy of predictions for different populations. The lack of sufficient empirical data on the pharmacokinetics of LTG under varying hormonal conditions and genetic backgrounds constrains the ability to model these effects accurately. This limitation is a significant factor in the difficulty of modeling this drug. It underscores the need for more extensive pharmacokinetic studies, especially in pregnant populations, incorporating detailed hormonal and genetic data.

By using a constant dose (200 mg), we established a baseline to compare how different populations (e.g., females vs. pregnant individuals) process the same amount of medication. This allows for clear observation of how pharmacokinetics differ due to physiological changes rather than differences in dosage. It helps in understanding the inherent variability in drug absorption, distribution, metabolism, and excretion between different populations without the confounding factor of dose variability, and it helps to identify how pregnancy-related physiological changes (e.g., increased blood volume, altered metabolism) impact drug levels and exposure. Since pregnancy can alter drug metabolism and clearance also, simulating varying doses throughout gestational weeks provides insight into how these changes affect drug exposure over time, leading to more accurate dosing recommendations. Understanding how drug exposure varies with different doses at various gestational stages allows for adaptive dosing strategies to maintain therapeutic efficacy and minimize side effects.

The model provides a useful tool for understanding how LTG pharmacokinetics change throughout pregnancy, offering a basis for adjusting doses to maintain therapeutic levels. For example, the model predicts a reduction in LTG plasma concentrations as pregnancy progresses, suggesting the need for dose adjustments to maintain efficacy and prevent suboptimal drug levels. By simulating different doses across gestational stages, the model helps in designing personalized dosing regimens that account for the physiological changes during pregnancy. This can guide clinicians in more accurately monitoring and adjusting doses, potentially improving treatment outcomes and minimizing adverse effects. Given the ethical and logistical challenges of including pregnant women in clinical trials, this model provides a strong argument for using observational data to simulate drug effects during pregnancy. Observational studies could enhance the model by providing real-world data on drug safety and efficacy in pregnant women, helping to refine dosing guidelines and improve patient care. Continuous refinement of the model, along with more comprehensive clinical data, will be essential for improving its accuracy and applicability in diverse patient populations.

### 4.2. EFV Model for Pregnant and Non-Pregnant Individuals: Clinical Considerations

The pharmacokinetics of EFV are notoriously variable, heavily influenced by genetic polymorphisms, particularly in the CYP2B6 gene [82], as well as by other factors such as gender. Drugs like EFV, which are primarily cleared hepatically, present challenges in simulation due to their complex metabolic pathways. Inhibitors or inducers of liver enzymes can interfere with EFV’s metabolism, significantly altering its pharmacokinetic profile. The inability of the current PBPK model to accurately predict the AUC and Cmax in certain populations underscores the complexity of these influencing factors.

The predicted Cmax values showed a slight overestimation at the 400 mg dose but an underestimation at the 600 mg dose, indicating dose-dependent discrepancies in the model. While these predictions fall within the observed variability reported in the literature, lending partial validity to the model, the significant underprediction at the higher dose highlights its limitations in accurately capturing EFV’s pharmacokinetics across all dosing conditions. Specifically, the model’s underestimation of AUC by 31.42% at the 600 mg dose in the general adult population suggests that, although the model reflects certain aspects of EFV pharmacokinetics, it does not fully account for all sources of variability.

During pregnancy, significant physiological changes occur that can alter drug metabolism, distribution, and excretion. The current PBPK model’s substantial underprediction of AUC (by −87.43% at 33 weeks’ gestation) and Cmax (by −66.39%) in pregnant women highlights the challenges of modeling EFV pharmacokinetics during pregnancy. These discrepancies likely arise from the model’s failure to fully incorporate the complex changes in enzyme activity, volume of distribution, and other physiological parameters that occur during pregnancy. While the predicted AUC and Cmax values fall within the broad ranges reported in clinical studies, the large percentage of errors indicate that the model does not reliably predict EFV exposure in pregnant populations. This limitation is further emphasized by the model’s performance at 23 weeks’ gestation, where the predicted AUC was −48.22% lower than observed, suggesting that even at earlier stages of pregnancy, the model struggles to accurately predict drug exposure. The slight improvement in Cmax prediction at 23 weeks (with a %PE of −3.22%) suggests that while the model may capture some aspects of peak plasma concentration, it still underestimates the overall drug exposure (AUC). This pattern of underprediction suggests that the model may not be adequately accounting for increased hepatic clearance or other pregnancy-related changes. The fact that the model does not consider genetic polymorphisms, particularly in CYP2B6, which significantly affects EFV metabolism, is a critical limitation. The variability in EFV clearance due to these genetic differences likely contributes to the discrepancies observed between predicted and observed pharmacokinetic parameters [43,83].

The observed differences in EFV pharmacokinetics between males and females, with higher AUC and Cmax values in males, align with reports in the literature that suggest sex-based differences in drug metabolism and exposure [84]. However, the inconsistency of these findings across studies suggests that more controlled research is needed to fully understand the influence of sex on EFV pharmacokinetics. Naidoo et al. [84] emphasize the need for more controlled studies to conclusively determine the influence of sex on EFV pharmacokinetics, due to mixed findings and potential confounding factors. For example, most women in the studies were of African origin, a population more likely to carry CYP2B6 alleles with reduced activity, and no genotyping was performed. Smith et al. [85] demonstrated the clinical implications of sex differences in treatment outcomes, particularly noting that women had a higher risk of virologic failure when treated with ATV/r compared to EFV. These findings suggest that sex-specific considerations are crucial in optimizing antiretroviral therapy, especially for women of childbearing potential, to ensure efficacy and minimize adverse effects. This underscores the need for gender-specific dosing guidelines to account for these differences.

The limitations of the current PBPK model are clear. It does not fully account for the complex physiological changes during pregnancy, including increased hepatic clearance, altered enzyme activity, and changes in the volume of distribution. Additionally, the model’s inability to incorporate genetic polymorphisms limits its predictive accuracy across diverse populations. The use of digitized data from published studies, while necessary, may introduce additional variability that could further complicate model accuracy, though this factor alone is unlikely to account for the large discrepancies observed.

Moving forward, it is imperative to refine the PBPK model to better account for pregnancy-specific physiological changes and genetic variability. This could involve incorporating more detailed patient-specific data, such as genotyping for CYP2B6, or using a population-based modeling approach that can better handle the wide variability seen in EFV pharmacokinetics. In vitro inhibition studies, particularly for enzymes like UGT2B7, are also needed to provide valuable insights into EFV metabolism and support more effective dosing strategies. Moreover, updating the GastroPlus model or developing new models that incorporate the complexities of pregnancy and genetic differences is crucial. This will enhance the clinical utility of these models, allowing for more accurate predictions of drug exposure and more personalized dosing strategies, particularly for vulnerable populations such as pregnant women.

### 4.3. Reduction in EFV’s Exposure Due to the Interaction with LTG

The observed reduction in Cmax and AUC for EFV under DDI conditions indicates that the interaction between these is minimal; the change in the total effect of EFV or LTG with co-administration was very small (Table 15 and Table 16). This suggests that the interaction might not be significant in terms of clinical outcomes. However, the study still emphasizes the importance of individualized dosing because even minimal interactions can be clinically relevant, especially in complex situations like HIV treatment and pregnancy.

EFV’s Cmax and AUC (0-inf) values slightly decrease when co-administered with LTG, and this reduction becomes more pronounced as gestational age progresses. However, the reduction is relatively modest and consistent across different stages of pregnancy. This suggests that while LTG might slightly enhance the metabolism or clearance of EFV, particularly in the later stages of pregnancy, the impact on EFV’s effectiveness may not be substantial enough to warrant significant clinical concern. These findings could play a supportive role in ensuring that EFV levels remain sufficient for therapeutic efficacy, particularly in clinical settings where EFV is a key component of treatment regimens for conditions like HIV. The interaction between LTG and EFV results in a slight but consistent increase in LTG’s Cmax and AUC across all gestational ages, indicating a modest enhancement in LTG exposure due to EFV co-administration. However, this increase is stable across different stages of pregnancy, suggesting that it is unlikely to require dose adjustments for LTG. The stability of LTG levels despite co-administration with EFV underscores the robustness of LTG dosing during pregnancy.

Further validation with clinical data is recommended to confirm these findings and guide appropriate dosing strategies, particularly in pregnant patients and those with different metabolic capacities (e.g., CYP and UGT metabolism). Additionally, exploring the limitations of the EFV PBPK model, especially regarding higher doses (600 mg), is warranted to ensure the model’s accuracy and reliability across various dosing scenarios.

## 5. Conclusions

This study utilized PBPK and p-PBPK models to simulate and predict the pharmacokinetics of LTG and EFV in pregnant women. The LTG model demonstrated a reduction in drug exposure due to physiological changes during pregnancy, highlighting the need for careful dose adjustments. However, the EFV model revealed complexities in accurately predicting pharmacokinetic changes during pregnancy. Although the model could simulate the overall trend of EFV exposure during pregnancy, discrepancies were observed between the predicted and observed data, indicating that the current model may require further refinement to fully capture the intricate dynamics of EFV metabolism in pregnant women. Despite these challenges, the study underscores the importance of developing and validating pregnancy-specific models to guide dosing decisions and improve therapeutic outcomes. The findings suggest that while the LTG model may be ready for clinical application with adjustments, the EFV model needs further calibration and validation before it can be reliably used in clinical practice. Therefore, continued research and refinement of these models are essential for optimizing drug therapy in pregnant women, particularly for medications like EFV that have complex pharmacokinetic profiles. This work contributes to the broader effort to ensure that pregnant women receive safe and effective medication management, ultimately enhancing maternal and fetal health outcomes.

## Figures and Tables

**Table 2:** Describes the rationale behind selecting specific doses for simulation at different gestational weeks to reflect clinical practice more accurately. To simulate each trimester in GastroPlus, we assigned a representative gestational week for each trimester and determined the corresponding hypothetical average dose. The dose selection is based on clinical usage patterns of lamotrigine reported in the literature, ensuring that the simulations are aligned with real-world clinical scenarios.

Trimester Simulated	Gestational Week	Hypothetical Average Dose	Rationale
First	10	150	Drug metabolism and clearance may be relatively similar to non-pregnant values during this period, so significant dose adjustments might not be necessary. The metabolic rate and clearance are close to pre-pregnancy levels so that a standard dose may suffice [25].
Second	20	200	Increased blood volume and renal clearance can lead to reduced drug levels. Therefore, dose adjustments may be required. The increased physiological changes necessitate a higher dose to maintain therapeutic levels [46].
Third	30	400	Significant drug metabolism and clearance changes often occur, which may require a higher dose to maintain therapeutic efficacy. Further increases in clearance and metabolism might necessitate a higher dose to achieve effective therapeutic levels [30,46].
Near delivery	40	300	Clearance rates may peak, potentially requiring dosage adjustments to avoid subtherapeutic levels. Further increases in clearance and metabolism might necessitate a higher dose to achieve effective therapeutic levels [46].

**Table 3:** LTG, enzyme table, and fm values for steady-state DDI predictions non-pregnant. The Vmax and Km values for non-pregnant individuals were collected from the literature [68]. For the different gestational weeks (10–40 weeks), the DDI module computed a Clint value (Clint = Vmax/K·m).

Non-Pregnant/Gestational Week	Enzyme	Vmax (mg/s/mg-Enzyme)	Km (µg/mL)	Clint (L/h)	fm (%)	Turnover (1/min)
Non-pregnant	UGT1A3	0.0000681	18.83	0.3411	8.63	-
	UGT1A4	0.00466	147.95	3.1564	79.90	-
10 weeks	UGT1A3	-	-	2.99 × 10^−1^	8.5	0.0005
	UGT1A4	-	-	2.77	78.65	0.0005
20 weeks	UGT1A3	-	-	2.88 × 10^−1^	8.46	0.0005
	UGT1A4	-	-	2.67	78.28	0.0005
30 weeks	UGT1A3	-	-	2.74 × 10^−1^	8.4	0.0005
	UGT1A4	-	-	2.53	77.73	0.0005
40 weeks	UGT1A3	-	-	2.61 × 10^−1^	8.34	0.0005
	UGT1A4	-	-	2.41	77.2	0.0005

**Table 4:** Enzyme EFV and preparator steady state DDI predictions. EFV as a moderate CYP3A4 inducer for DDI predictions using available induction parameters and observations.

Enzyme	CYP2B6	CYP3A4	UGT1A4	UGT1A9
Vmax (mg/s/mg-enzyme)	0.050 (PBPK)	0.000562 (PBPK), 0.2 (Gut)	0.0000208 (PBPK), 0.0583 (Liver)	0.000111 (PBPK), 0.0091 (Liver)
Km (µg/mL)	3.137	3.137	0.069	0.0051
Inh/Ind Const Value (µM)	0.82	1	2	9.4
Inh/Ind Const Type	EC50-in vitro, T (induction)	EC50-in vitro, T	Ki-rev-in vitro T	Ki-rev-in vitro T
Emax	5.76	9.9	-	-
In vitro Fu	0.063	0.063	0.12	0.12
In vitro Fu Type	User	User	Calc(Austin)-HLM	Calc(Austin)-HLM
In vitro Prot Conc (mg/mL)	0	0	0.5	0.5
Ref	GastroPlus model standard	GastroPlus model standard	[17]	[17]

**Table 5:** Model validation results: pharmacokinetic parameters predicted (Pred) and observed (Obs) for oral administration of lamotrigine in adults, for different doses.

Dose	Dosage Form/Protocol	AUC 0–t (μg/mL-h)			Cmax μg/mL			Tmax (h)		
		Pred	Obs	%PE	Pred	Obs	%PE	Pred	Obs	%PE
25 mg Ebert et al. [70]	Oral/SD	8.42	7.9	6.64	0.31	0.26	18.27	1.6	2.65	−39.62
75 mg Peck et al. [69]	Oral/SD	25.85	23.30	10.92	1.03	0.883	17.11	1.1	2.108	−47.82
100 mg van Luin et al. [71]	Oral/SD	33.81	33.8	0.07	1.37	1.13	20.89	1.14	2.13	−47.8
200 mg Incecacyir et al. [72]	Oral/SD	91.56	106.91	−14.35	2.75	2.84	−3.13	1.1	1.77	−37.99
200 mg Wootton et al. [73]	Oral/SD	93.001	89.027	4.470	2.74	2.36	16.01	1.1	2.538	−56.66

**Table 6:** Model validation: pharmacokinetic parameters predicted (Pred) and observed (Obs) for oral administration of lamotrigine during pregnancy.

Dose	Dosage Form/Protocol	AUC 0–t (μg/mL-h)			Cmax μg/mL			Tmax (h)		
400 mg Reimers et al. [29]	Oral/SD	Pred	Obs	%PE	Pred	Obs	%PE	Pred	Obs	%PE
		47.67	43.4	9.84	5.05	5.3	−4.064	1.32	2.3	−42.6

**Table 7:** Statistical metrics for the evaluation of Standard (Adult male) and Pregnant LTG PBPK model.

Metric	Standard Cmax	Standard AUC	Pregnancy Cmax	Pregnancy AUC
AFE	1.1423	0.913	0.923	1.098
AAFE	0.9130	1.0953	1.049	1.098
MAE	0.35	9.47	0.25	4.3
RMSE	0.35	9.47	0.25	4.3

**Table 8:** Simulate LTG at a constant dose (e.g., 200 mg) across all gestational weeks.

	F (%)	Cmax (µg/mL)	Tmax (h)	AUC 0–24 (µg-h/mL)	AUC 0-inf (µg-h/mL)	Cmax Liver (µg/mL)
Ault standard model	97.845	2.75	1.12	44.367	93.271	5.733
Female	97.99	2.96	0.72	46.145	103.62	6.82
10 GA	97.968	2.88	0.8	44.92	100.31	6.68
20 GA	97.91	2.79	0.88	43.50	95.23	6.64
30 GA	97.85	2.68	0.96	41.934	41.934	6.33
40 GA	97.74	2.41	1.04	38.454	90.72	6.61

**Table 9:** Simulation of varying dosing of LTG in the different populations and gestational ages for 24 h. This provides a baseline to compare how varying the dose across weeks affects PK.

	Dose	F (%)	Cmax (µg/mL)	Tmax (h)	AUC 0–24 (µg-h/mL)	AUC 0-inf (µg-h/mL)	Cmax Liver (µg/mL)
Ault standard model	100	97.94	1.59	0.72	22.86	49.67	3.72
Female	100	97.98	1.51	0.64	23.07	51.63	3.78
10 GA	150	97.96	2.19	0.72	33.69	75.10	5.26
20 GA	200	97.91	2.80	0.88	43.50	95.23	6.64
30 GA	400	97.87	5.11	1.28	83.92	183.45	12.331
40 GA	300	97.75	3.52	1.28	57.68	136.55	9.49

**Table 11:** Model validation: pharmacokinetic parameters predicted (Pred) and observed (Obs) for oral administration of EFV during pregnancy.

Dosage Form and GA	Dose	AUC 0–t (µg/mL-h)			Cmax µg/mL			Tmax		
		Pred	Obs	%PE	Pred	Obs	%PE	Pred	Obs	%PE
Oral/SD—33 weeks	600 mg Cressey et al. [26]	20.77	165.18	−87.4283	2.79	8.33	−66.39	1.36	1.94	−30.04
Oral/SD—23 weeks	600 mg Lartey et al. [33]	22.27	43.00	−48.22	2.99	3.09	−3.22	1.28	3.61	−64.51

**Table 12:** Statistical metrics for the evaluation of Standard (Adult male) and Pregnant EFV PBPK model.

Metric	Standard Cmax	Standard AUC	Pregnancy Cmax	Pregnancy AUC
AFE	1.23	1.21	0.28	0.43
AAFE	1.23	1.21	3.44	2.35
MAE	0.9	11.84	3.13	30.96
RMSE	0.9	11.84	3.13	30.96

**Table 13:** Comparison of different doses (600 and 400 mg) in different PBPK models, with the PK parameters adjusted for a male adult (standard) and a female adult.

PBPK Model	Dose	F (%)	Cmax (µg/mL)	Tmax (h)	AUC 0–24 (µg-h/mL)	AUC 0-inf (µg-h/mL)	Cmax Liver (µg/mL)
Standard	400	88.43	2.72	1.6	32.54	117.09	12.92
Female	400	82.91	2.17	1.12	17.29	24.95	11.35
Standard	600	90.17	4.14	1.6	49.75	179.12	19.56
Female	600	85.63	3.38	1.2	27.92	40.87	17.56

**Table 14:** Simulation of 600 mg of EFV for 24 h. Simulation of female and pregnant PBPK model.

	F (%)	Cmax (µg/mL)	Tmax (h)	AUC 0–24 (µg-h/mL)	AUC 0-inf (µg-h/mL)	Cmax Liver (µg/mL)
Female	85.63	3.38	1.2	27.92	40.87	17.56
10 GA	83.24	3.19	1.2	25.29	35.9	17.22
20 GA	81.44	3.04	1.28	22.914	31.35	17.34
30 GA	80.38	2.88	1.35	21.23	28.43	17.63
40 GA	79.67	2.58	1.36	19.85	27.53	17.96

**Table 15:** Ration obtained based on the dynamic simulation of drug-drug interactions between LTG and EFV across gestational ages.

PBPK Model	Drug	Fa [%]	FDp [%]	F [%]	Cmax [µg/mL]	Tmax [h]	AUC (0-t) [µg-h/mL]	AUC (0-inf) [µg-h/mL]
Female (100 mg)	LTG	1	1	1.001	1.003	1	0.001009	0.001016
	EFV	1	0.976	0.973	0.991	0.933	0.000953	0.000934
10 GA (150 mg)	LTG	1	1	1.001	1.003	1	0.001009	0.001013
	EFV	1	0.969	0.964	0.988	1	0.000945	0.000925
20 GA (200 mg)	LTG	1	1	1.001	1.004	1	0.001009	0.001013
	EFV	1	0.963	0.958	0.987	0.938	0.000939	0.000923
30 GA (400 mg)	LTG	1	1	1.001	1.004	1	0.001008	0.001016
	EFV	1	0.961	0.955	0.986	0.941	0.000934	0.000919
40 GA (300 mg)	LTG	1	1	1.002	1.004	1	0.001007	0.001007
	EFV	1	0.963	0.956	0.985	1	0.000934	0.000916

**Table 16:** Results of the Dynamic Simulation Assessing Drug-Drug Interactions Between LTG and EFV in the Standard PBPK Model.

PBPK Model—Dose	Drug	Fa [%]	FDp [%]	F [%]	Cmax [μg/mL]	Tmax [h]	AUC (0-t) [µg-h/mL]	AUC (0-inf) [µg-h/mL]
Adult (standard)—200 mg	LTG	1	1	1.001	1.003	1	0.001013	0.00103
Adult (standard)—400 mg	EFV	1	0.986	0.986	0.994	0.958	0.000985	0.000865

## Data Availability

The original contributions presented in the study are included in the article/Supplementary Material. Further inquiries can be directed to the corresponding author.

## Supplementary Materials

The following supporting information can be downloaded at: https://www.mdpi.com/article/10.3390/pharmaceutics16091163/s1, Table S1. Summary of Studies Utilized for LTG PBPK and Pregnant PBPK Model Development and Validation; Figure S1. Predicted and observed mean plasma concentration-time profiles of orally administered tablets of lamotrigine. The solid line represents the predicted mean. Circles are the mean observed data for the different doses: (A) 25 mg, (B) 75 mg, (C) 100 mg, (D) 200 mg, (E) 200 mg. Table S2. Summary of Studies Utilized for EFV PBPK and Pregnant PBPK Model Development and Validation. Figure S2. Predicted and observed mean plasma concentration-time profiles of orally administered tablets of efavirenz. The solid line represents the predicted mean. Circles are the mean observed data from (A), (B) at 600 mg. Table S3. Results of the Dynamic Simulation Assessing Drug-Drug Interactions Between LTG and EFV in the Female PBPK Model and the Pregnant Model Across Gestational Ages. Table S4. Results of the Dynamic Simulation Assessing Drug-Drug Interactions Between LTG and EFV in the Standard PBPK Model.
