# Supplementary material for: PBPK Modeling of Lamotrigine and Efavirenz during Pregnancy: Implications for Personalized Dosing and Drug-Drug Interaction Management
Source: Pharmaceutics. 2024 Sep 3;16(9):1163. doi: 10.3390/pharmaceutics16091163 (PMC11435310; doi:10.3390/pharmaceutics16091163)
Supplement: Supplementary file 1 [file pharmaceutics-16-01163-s001.zip › pharmaceutics-3121704-supplementary.pdf]

Supplementary materials

Table S1. Summary of Studies Utilized for LTG PBPK and Pregnant PBPK Model Development and Validation.

Population	Age (years)	Gestational Week	Dose (mg)	n	Route/ regimen	Cmax (µg/mL)	AUC 0-t (µg-h/mL)	Reference
Non-pregnant	25 +/- 4	-	25	10	SD	0.29 ± 0.02	7.95 ± 0.75	Ebert et al.
	21-48	-	75	8	Oral/SD	1.56 ± 0.33	-	Peck et al.
	35-59	-	100	12	Oral/SD	1.28 (1.20 - 1.37)	33.1 (31.3 - 35.0)	Van Luin et al.
	23 ± 2	-	200	14	Oral/SD	2.91 ± 0.26	123 ± 14	Incecayir et al.
	35-57	-	200	12	Oral/SD	2.46 ± 0.41	94.6 ± 21.6	Wooton et al. **X**
Pregnant	21-32	0 to birth	Various	14	Mono or Polytherapy/SD and MD	-	-	Tran et al.
	17-42	0 to birth	426.81	14	No specific information – cohort analysis	-	-	Penell et al.
	17-39	Different times of enrolment and variable ability to participate	400	19	No specific information	5.3 ± 4.3	43.4 ± 34.2	Reimers et al.

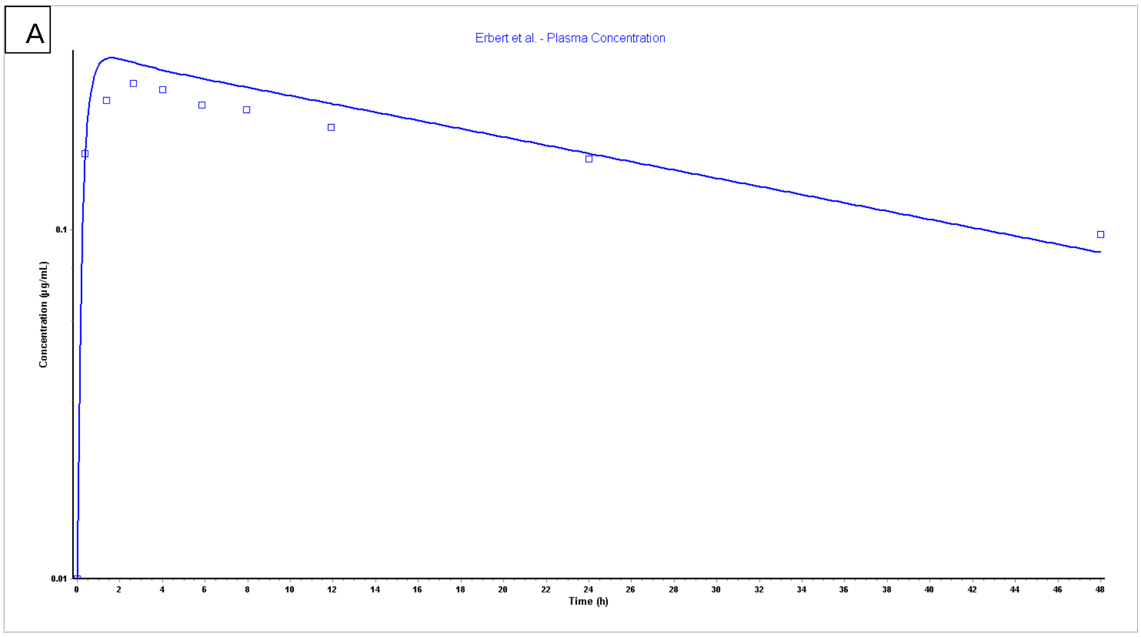
A
Erbert et al. - Plasma Concentration
Concentration (µg/mL)
0.1
0.01
0 2 4 6 8 10 12 14 16 18 20 22 24 26 28 30 32 34 36 38 40 42 44 46 48
Time (h)

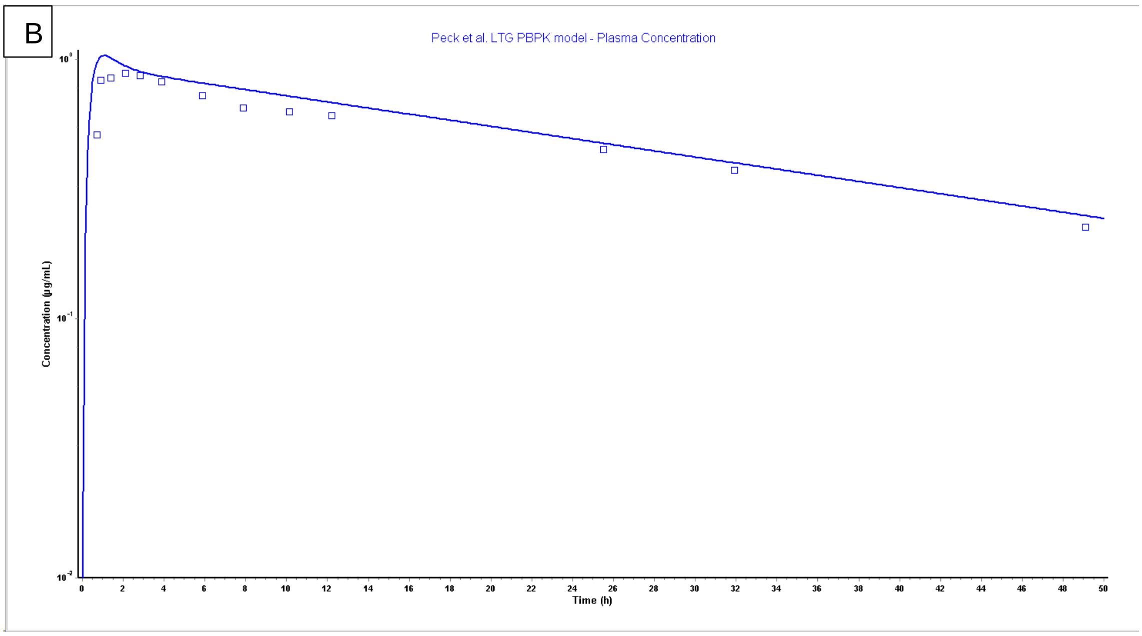
B
Peck et al. LTG PBPK model - Plasma Concentration
Concentration (µg/mL)
0 2 4 6 8 10 12 14 16 18 20 22 24 26 28 30 32 34 36 38 40 42 44 46 48 50
Time (h)

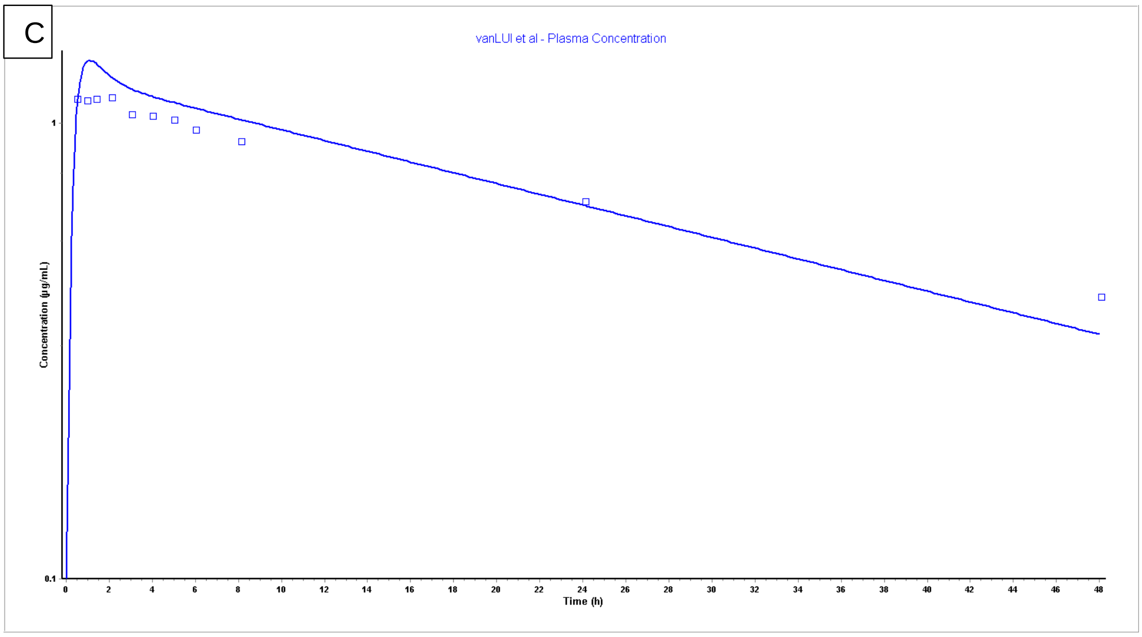
C
vanLUI et al - Plasma Concentration
Concentration (µg/mL)
1
0.1
0 2 4 6 8 10 12 14 16 18 20 22 24 26 28 30 32 34 36 38 40 42 44 46 48
Time (h)

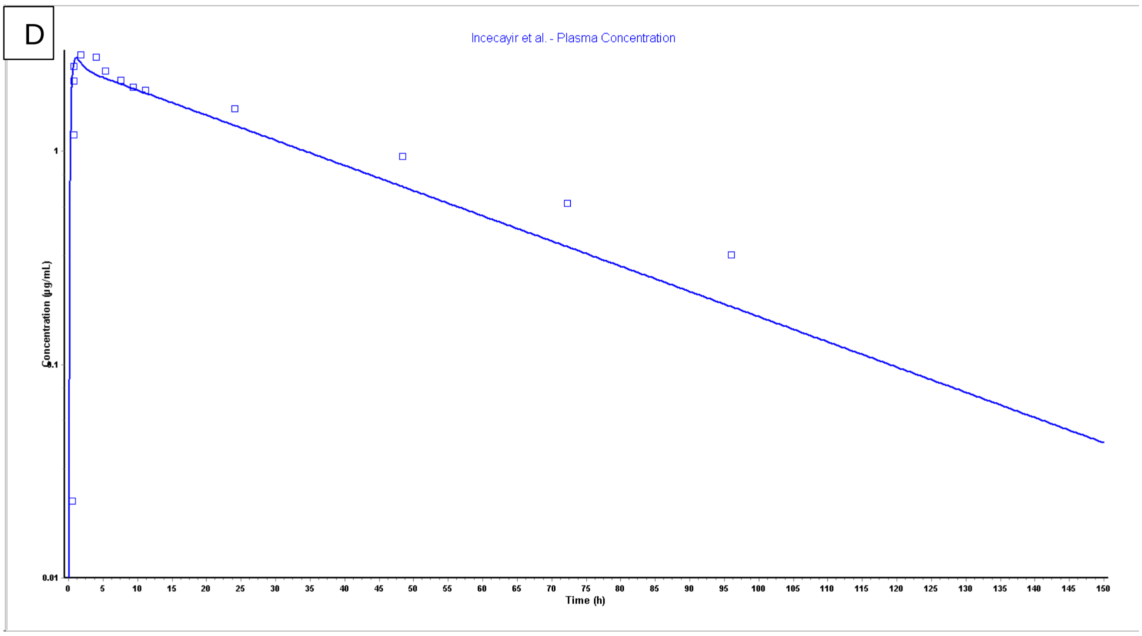
D
Incecayir et al. - Plasma Concentration
Concentration (µg/mL)
1
0.1
0.01
0 5 10 15 20 25 30 35 40 45 50 55 60 65 70 75 80 85 90 95 100 105 110 115 120 125 130 135 140 145 150
Time (h)

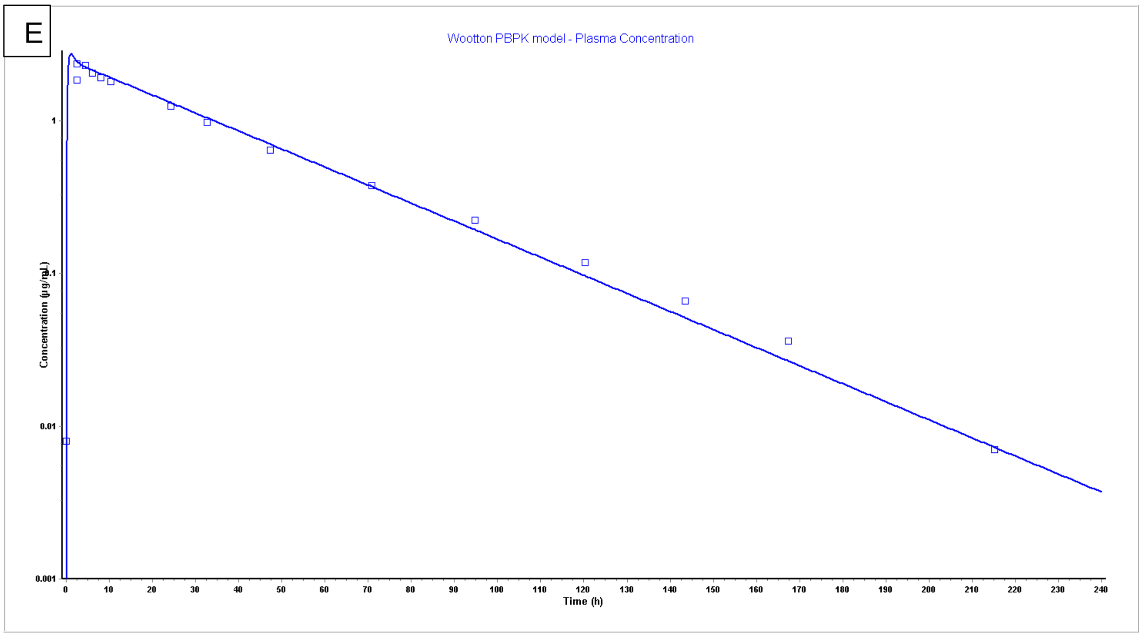

Figure S1. Predicted and observed mean plasma concentration-time profiles of orally administered tablets of lamotrigine. The solid line represents the predicted mean. Circles are the mean observed data for the different doses: (A) 25 mg, (B) 75 mg, (C) 100 mg, (D) 200 mg, (E) 200 mg.

Table S2. Summary of Studies Utilized for EFV PBPK and Pregnant PBPK Model Development and Validation.

Population	Age (years)	Gestational Week	Dose (mg)	n	Route/ regimen	Cmax (µg/mL)	AUC 0-t (µg-h/mL)	Reference
Non-pregnant	-	-	400<br>600	636	Oral/SD	2.52 (2.42-2.62)<br>3.66 (3.51-3.81)	49.2 (47.0-51.5)<br>67.2 (63.8-70.9)	Dickinson et al.
	22-60	-	600	44	Oral/BID	4.0 ± 1.71	57.15 ± 27.3	Villany et al.
	41-56	-	600	82	Oral/BID			Ximenez et al.
	22-60	-	400	22	SD	3.257 (2.554 - 4.1454)	52.259 (38.284 – 71.335)	Cerrone et al.
	23-32	-	400	20	SD			Xu et al.
Pregnant	18-42	30-39	600	25	SD	5.44 (1.90 – 12.22)	55.4 (13.5 – 220.3)	Cressey et al.
	22-34	8-32	600	25	SD	4.33 (3.42 – 5.48)	52.32 (39.07 - 70.6)	Lartey et al.
	NA	21-39	600	258	NA	-	-	Schalkwijk et al.
	25-31	36-40	600	97	SD	-	-	Dooley et al.

NA – not available

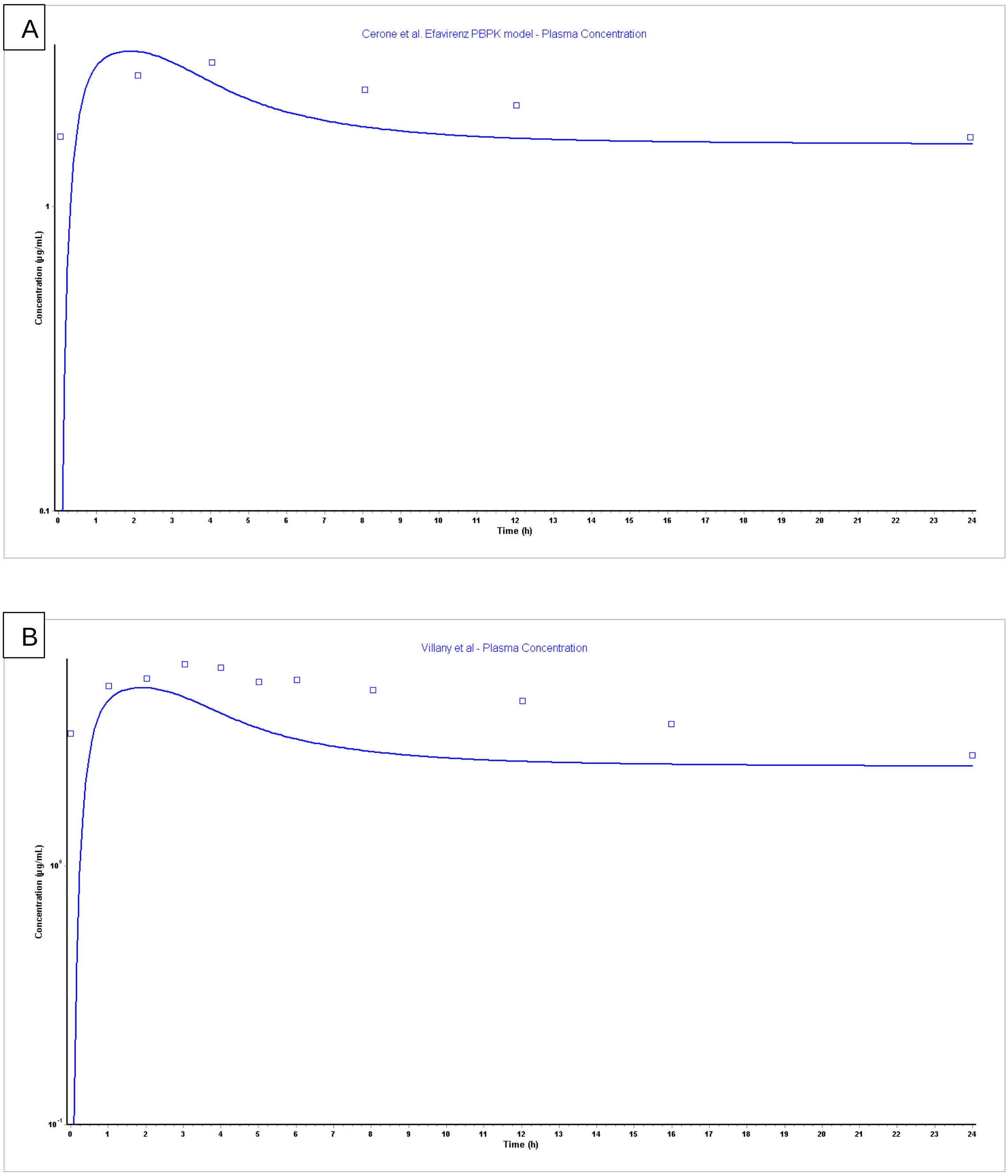

Figure S2. Predicted and observed mean plasma concentration-time profiles of orally administered tablets of efavirenz. The solid line represents the predicted mean. Circles are the mean observed data from (A) , (B) at 600 mg.

Table S3. Results of the Dynamic Simulation Assessing Drug-Drug Interactions Between LTG and EFV in the Female PBPK Model and the Pregnant Model Across Gestational Ages.

**PBPK model**	**Drug**	**Condition**	**Fa [%]**	**FDp [%]**	**F [%]**	**Cmax [ug/mL]**	**Tmax [h]**	**AUC(0-t) [μg-h/mL]**	**AUC(0-inf) [μg-h/mL]**
Female	LTG	Baseline	99.75	99.65	97.98	1.514	0.64	23.1	51.6
	EFV		99.99	95.12	85.63	3.38	1.2	27.9	40.9
	LTG	DDI	99.75	99.64	98.11	1.518	0.64	23.3	52.4
	EFV		99.99	92.87	83.29	3.348	1.12	26.6	38.2
	LTG	Ratio	1	1	1.001	1.003	1	0.001009	0.001016
	EFV		1	0.976	0.973	0.991	0.933	0.000953	0.000934
10 GA	LTG	Baseline	99.75	99.65	97.96	2.189	0.72	33.7	75.1
	EFV		99.99	93.72	83.24	3.195	1.2	25.3	35.9
	LTG	DDI	99.75	99.65	98.09	2.196	0.72	34	76.1
	EFV		99.99	90.78	80.27	3.158	1.2	23.9	33.2
	LTG	Ratio	1	1	1.001	1.003	1	0.001009	0.001013
	EFV		1	0.969	0.964	0.988	1	0.000945	0.000925
20 GA	LTG	Baseline	99.76	99.66	97.91	2.799	0.88	43.5	95.2
	EFV		100	92.94	81.44	3.039	1.28	22.9	31.3
	LTG	DDI	99.75	99.65	98.04	2.809	0.88	43.9	96.4
	EFV		100	89.53	78.03	3.001	1.2	21.5	28.9
	LTG	Ratio	1	1	1.001	1.004	1	0.001009	0.001013
	EFV		1	0.963	0.958	0.987	0.938	0.000939	0.000923

30 GA	LTG	Baseline	99.76	99.65	97.87	5.111	1.28	83.9	183
	EFV		100	92.91	80.38	2.876	1.36	21.2	28.4
	LTG	DDI	99.75	99.65	98.01	5.132	1.28	84.6	186
	EFV		100	89.28	76.76	2.836	1.28	19.8	26.1
	LTG	Ratio	1	1	1.001	1.004	1	0.001008	0.001016
	EFV		1	0.961	0.955	0.986	0.941	0.000934	0.000919
40 GA	LTG	Baseline	99.76	99.66	97.75	3.522	1.28	57.7	137
	EFV		100	93.76	79.67	2.584	1.36	19.8	27.5
	LTG	DDI	99.76	99.66	97.9	3.536	1.28	58.1	138
	EFV		100	90.28	76.14	2.546	1.36	18.5	25.2
	LTG	Ratio	1	1	1.002	1.004	1	0.001007	0.001007
	EFV		1	0.963	0.956	0.985	1	0.000934	0.000916

GA: Gestational Age; Fa: Fraction absorbed; FDp: Fraction dose absorbed (portal vein); F: Bioavailability; Cmax: Maximum plasma concentration; Tmax: Time to reach Cmax; AUC(0-t): Area under the plasma concentration-time curve from time zero to the last measurable concentration; AUC(0-inf): Area under the plasma concentration-time curve from time zero to infinity

Table S4. Results of the Dynamic Simulation Assessing Drug-Drug Interactions Between LTG and EFV in the Standard PBPK Model.

**PBPK model - Dose**	**Drug**	**Condition**	**Fa [%]**	**FDp [%]**	**F [%]**	**Cmax [ug/mL]**	**Tmax [h]**	**AUC(0-t) [µg-h/mL]**	**AUC(0-inf) [µg-h/mL]**
Adult (standard) – 200 mg	LTG	Baseline	99.75	99.64	97.85	3.065	0.72	44.6	93.3
Adult (standar	EFV	Baseline	99.99	96.51	96.12	3.242	1.92	45.6	1260

d) – 400 mg								
Adult (standard) – 200 mg	LTG	DDI	99.74	99.64	97.96	3.073	0.72	45.2	96.1
Adult (standard) – 400 mg	EFV	DDI	99.99	95.16	94.78	3.221	1.84	44.9	1090
Adult (standard) – 200 mg	LTG	Ratio	1	1	1.001	1.003	1	0.001013	0.00103
Adult (standard) – 400 mg	EFV	Ratio	1	0.986	0.986	0.994	0.958	0.000985	0.000865
